# Association between serum zinc levels and new-onset hypertension: A 5-year retrospective cohort study

**DOI:** 10.1097/MD.0000000000050885

**Published:** 2026-09-25

**Authors:** Kuo-Chuan Hung, Hsiu-Lan Weng, Shu-Wei Liao, Yi-Chen Lai, Ming-Chung Lin, I-Wen Chen

**Affiliations:** a Department of Anesthesiology, Chi Mei Medical Center, Tainan, Taiwan; b School of Medicine, College of Medicine, National Sun Yat-sen University, Kaohsiung, Taiwan; c Department of Anesthesiology, E-Da Hospital, I-Shou University, Kaohsiung, Taiwan; d Center of General Education, Chia Nan University of Pharmacy and Science, Tainan, Taiwan; e Department of Anesthesiology, Chi Mei Hospital, Liouying, Tainan, Taiwan.

**Keywords:** cardiac, hypertension, hypertensive crisis, renal, zinc

## Abstract

The relationship between zinc deficiency and incident hypertension remains poorly characterized in humans. We examined the association between zinc deficiency and new-onset hypertension and related complications. This retrospective cohort study utilized the TriNetX Research Network to analyze adult patients with serum zinc measurements between January 2010 and December 2023. The zinc-deficient group had concentrations below 0.7 µg/mL, while controls ranged from 0.70 to 1.20 µg/mL. Following propensity score matching, 47,843 pairs were analyzed over 5 years. The primary outcome was new-onset primary hypertension. Secondary outcomes included secondary hypertension, hypertension-related cardiac and renal complications, and hypertensive crisis. Zinc deficiency was significantly associated with primary hypertension (hazard ratio [HR] 1.20, 95% confidence interval [CI] 1.14–1.25; *P* < .001), secondary hypertension (HR 1.82, 95% CI 1.45–2.29; *P* < .001), hypertension-related cardiac and renal complications (HR 1.69, 95% CI 1.49–1.92; *P* < .001), and hypertensive crisis (HR 1.09, 95% CI 1.01–1.18; *P* = .028). Dose–response analyses revealed a J-shaped association for primary hypertension, with elevated hazards at both low and high zinc concentrations. The association between zinc deficiency and primary hypertension was significantly stronger in men than in women (HR 1.28 vs 1.13; *P* for interaction = .021). Zinc deficiency was associated with incident hypertension and related complications, particularly secondary hypertension. Further prospective studies are warranted to determine whether correction of zinc deficiency modifies hypertension risk.

## 1. Introduction

Hypertension affects over 1 billion individuals worldwide and remains the leading modifiable risk factor for cardiovascular disease, stroke, and premature mortality.^[1–3]^ Despite the availability of multiple antihypertensive agents, blood pressure control rates remain suboptimal,^[4,5]^ highlighting the critical need to identify novel preventable risk factors. Recent nutritional epidemiologic studies have reported nonlinear associations between the intake of several micronutrients, including copper, iron, vitamin A, and vitamin E, and the risk of new-onset hypertension,^[6–9]^ suggesting that micronutrient imbalance may play a crucial role in hypertension pathogenesis. Zinc is an essential trace element involved in over 300 enzymatic reactions and plays crucial roles in antioxidant defense, vascular function, and cardiovascular homeostasis.^[10–13]^ Mechanistic studies have established a causal link between zinc deficiency and hypertension in animal models. Williams et al demonstrated that zinc-deficient mice developed hypertension through upregulation of the sodium–chloride cotransporter in the distal convoluted tubule, promoting excessive renal sodium reabsorption.^[14]^ Subsequent investigations confirmed that zinc deficiency amplifies salt sensitivity and activates multiple sodium transporters, including the epithelial sodium channel.^[15]^ These experimental findings provide compelling biological plausibility for the role of zinc in blood pressure homeostasis.

However, clinical evidence linking zinc deficiency to new-onset hypertension in humans remains inconsistent and incomplete. A meta-analysis of observational studies reported significantly lower serum zinc levels in hypertensive patients compared with controls,^[16]^ while a meta-analysis of randomized controlled trials showed that zinc supplementation modestly reduced systolic blood pressure by approximately 1.5 mm Hg.^[17]^ Although these findings suggest a potential association between zinc status and blood pressure regulation, these studies primarily examined individuals with established hypertension rather than evaluating the impact of zinc levels on the risk of developing new-onset hypertension. Furthermore, while a small case-control study reported an association between low serum zinc levels and prehypertension,^[18]^ a large prospective cohort study^[19]^ found that higher serum zinc concentrations were associated with an increased risk of hypertension, contrasting with several previous experimental and observational findings.

To address these critical knowledge gaps, we conducted a large-scale, longitudinal cohort study using the TriNetX Research Network to investigate the association between zinc deficiency and the risk of new-onset hypertension. Given the observational nature of the study, our objective was not to establish a causal relationship but to evaluate whether zinc deficiency was associated with a higher subsequent risk of incident hypertension. We further examined whether this association varied across serum zinc concentrations and according to age and sex. We hypothesized that zinc deficiency would be associated with an increased risk of incident hypertension, with stronger associations at lower zinc concentrations.

## 2. Methods

### 2.1. Data source

This retrospective cohort study utilized data from the TriNetX Research Network, a global federated health research platform that aggregates de-identified electronic medical records from participating healthcare organizations. TriNetX has been widely used in peer-reviewed real-world evidence studies across diverse clinical disciplines.^[20–22]^ The database encompasses comprehensive real-world data, including demographic information, diagnoses, procedures, medications, and laboratory results, standardized using common data models and terminologies such as International Classification of Diseases, 10th revision, Clinical Modification, Logical Observation Identifiers Names and Codes, Current Procedural Terminology, and Anatomical Therapeutic Chemical codes. All data within TriNetX were de-identified in compliance with the Health Insurance Portability and Accountability Act. The study protocol was approved by the Institutional Review Board of Chi Mei Medical Center (IRB number:11310-E04), which granted a waiver for informed consent in compliance with observational research regulations, as this analysis involved only aggregated, anonymized data.

### 2.2. Study population and cohort definition

Adult patients aged ≥18 years with available serum zinc measurements between January 1, 2010, and December 31, 2023, were eligible for inclusion. The zinc-deficient group included patients with serum zinc concentrations <0.7 µg/mL (<70 µg/dL), an operational threshold used to define zinc deficiency or hypozincemia in previous adult clinical studies,^[23–25]^ whereas the control group comprised patients with zinc levels between 0.70 and 1.20 µg/mL. Because fasting status and the exact time of blood collection were not consistently available in the TriNetX data, serum zinc measurements could not be standardized according to fasting status or sampling time. The date of zinc measurement served as the index date for subsequent follow-up.

To ensure that the analysis focused on incident hypertension, patients with any documented diagnosis of essential hypertension (I10), secondary hypertension (I15), gestational hypertension (O14–O16), or unspecified hypertensive disorders (I10–I1A) before the index date were excluded from both cohorts. Patients were also excluded if they had chronic kidney disease stage 4 (N18.4), stage 5 (N18.5), or end-stage renal disease (N18.6) prior to the zinc test, as severe renal dysfunction may alter zinc metabolism and confound its association with blood pressure regulation. To minimize confounding from acute illness or inflammation, patients with acute kidney failure (N17), hospitalization or intensive care unit admission, or C-reactive protein ≥10 mg/L within 1 month before the index date were excluded. Patients with endocrine or neuroendocrine disorders known to cause secondary hypertension, including Conn syndrome (E26.01), Cushing disease (E24.0), or neoplasms of the endocrine glands (e.g., pheochromocytoma) (D49.7), were also excluded.

### 2.3. Data collection and propensity score matching

Baseline characteristics collected at the index date included age, sex, race, body mass index, and comorbidities such as diabetes mellitus, obesity, dyslipidemia, chronic kidney disease, liver disease, malnutrition, sleep disorders, and mood disorders. Laboratory parameters included hemoglobin, serum albumin, glycated hemoglobin, and estimated glomerular filtration rate. Medication histories were reviewed for micronutrient supplementation, including zinc, vitamin D, and overall vitamin supplementation.

To minimize baseline differences between the zinc-deficient and control cohorts, propensity score matching was performed using the built-in TriNetX analytics module. Propensity scores were estimated using logistic regression based on the demographic characteristics, clinical comorbidities, and laboratory data available at baseline. Covariates were selected a priori based on their potential associations with serum zinc status and/or hypertension risk and included demographic factors, established cardiovascular and metabolic risk factors, renal and hepatic conditions, indicators of nutritional status, relevant laboratory parameters, and vitamin supplementation. However, detailed dietary intake and certain lifestyle and socioeconomic variables were not consistently available in the database and therefore could not be incorporated into the matching model. A one-to-one greedy nearest-neighbor matching algorithm with a caliper width of 0.1 standard deviation of the logit of the propensity score was used. These matching parameters are predefined within the TriNetX analytics workflow available for this study and cannot be modified by investigators to apply alternative caliper widths or matching algorithms. Standardized mean differences (SMD) were calculated to assess covariate balance, with values below 0.1 considered indicative of negligible residual imbalance. Visual confirmation of the propensity score distribution before and after matching was performed to ensure appropriate overlap between cohorts.

### 2.4. Outcomes

The primary outcome was new-onset hypertension (I10) within 5 years after the index date, defined as the 1st recorded diagnosis of essential hypertension following cohort entry. Secondary outcomes included secondary hypertension (I15), hypertension-related cardiac or renal complications (I11–I13), and hypertensive crisis identified by diagnosis codes (I16) or blood pressure readings of ≥180 mm Hg systolic or ≥120 mm Hg diastolic. For diagnosis-based outcomes, the 1st qualifying diagnosis recorded during follow-up was considered the incident event.

Pneumonia was included as a positive control outcome because of the well-established association between zinc deficiency and impaired immune function and infection susceptibility, allowing us to assess whether the analytical framework reproduced an expected exposure–outcome association. To reduce the likelihood that early diagnoses represented preexisting but previously undocumented disease, a 3-month washout period was applied to all study outcomes; only events 1st recorded 3 months or later after the index zinc measurement were included.

### 2.5. Sensitivity and subgroup analyses

We assessed outcomes at 3- and 7-year follow-up intervals to evaluate the consistency of the findings across different follow-up durations. Subgroup analyses were conducted to investigate whether the association between zinc levels and outcomes varied across different patient populations stratified by age (18–50 vs >50 years) and sex.

### 2.6. Dose–response analysis

To examine potential dose–response relationships, we created 4 cohorts based on zinc levels: <0.5, 0.5 to 0.7, 0.7 to 1.2, and >1.2 µg/mL. The threshold of 0.7 µg/mL was selected as the operational definition of zinc deficiency based on its use in previous adult clinical studies,^[23–25]^ while 0.7 to 1.2 µg/mL was used as the reference range. Within the deficient range, <0.5 µg/mL was used to identify more severe zinc deficiency, consistent with previous clinical categorizations,^[26]^ whereas values >1.2 µg/mL represented concentrations above the reference range. These prespecified categories allowed assessment of whether associations differed across low, reference, and elevated zinc concentrations. Each zinc-level cohort was propensity score-matched to the control group and analyzed using the same methods as the primary analysis.

### 2.7. Statistical analysis

All statistical analyses were conducted using the built-in analytics modules of the TriNetX Research Network platform. Continuous variables were summarized as means with standard deviations, and categorical variables were expressed as counts and percentages. Outcomes were evaluated using time-to-event analysis. Cumulative incidence was estimated using Kaplan–Meier survival curves, and inter-group differences were assessed using the log-rank test. Hazard ratios (HRs) with corresponding 95% confidence intervals were calculated using Cox proportional hazards models, with the control cohort serving as the reference group. The proportional hazard assumption was verified using a test for proportionality within TriNetX. All analyses were performed on propensity score-matched cohorts to minimize confounding bias. No additional multivariable covariate adjustment was performed in the Cox models because measured confounding was addressed through propensity score matching before outcome analysis. For subgroup analyses, *P* values for interaction were calculated by comparing the subgroup-specific log HRs and their standard errors to assess whether the magnitude of the association differed between age or sex strata. These *P* values therefore represent between-subgroup differences in effect estimates rather than interaction terms directly fitted within the Cox models.

## 3. Result

### 3.1. Patient selection and baseline characteristics

From the TriNetX Research Network, 382,177 adults with serum zinc measurements between 2010 and 2023 were identified. After exclusions, 48,454 patients comprised the zinc-deficient cohort and 115,676 the control cohort. One-to-one propensity score matching yielded 47,843 well-balanced pairs (Fig. 1). Before matching, both cohorts had relatively low comorbidity burdens. One-to-one propensity score matching yielded 47,843 matched pairs with excellent covariate balance (all SMD < 0.1). Table 1 presents the distributions and SMDs of all matching covariates both before and after propensity score matching. Several variables showed SMDs > 0.1 before matching; however, all measured covariates achieved SMDs < 0.1 after matching. Post-matching, both cohorts had comparable age (43.7 ± 18.0 vs 43.2 ± 17.6 years), sex distribution (24.7% vs 22.8% male), and comorbidity profiles. All baseline characteristics, laboratory parameters, and medication history were adequately balanced (Table 1). Propensity score distributions demonstrated an appropriate overlap between cohorts after matching (Fig. 2).

**Table 1:** Baseline characteristics of patients with and without zinc deficiency before and after propensity score matching.

Variables	Before matching			After matching		
	ZD group (n = 48,454)	Control group (n = 115,676)	SMD*	ZD group (n = 47,843)	Control group (n = 47,843)	SMD*
Patient characteristics						
Age at index (yr)	43.8 ± 18.0	42.4 ± 17.2	0.078	43.7 ± 18.0	43.2 ± 17.6	0.031
Male (%)	12,001 (24.8)	33,652 (29.1)	0.098	11,818 (24.7)	10,887 (22.8)	0.046
BMI (kg/m^2^)	28.5 ± 9.1	28.5 ± 8.6	0.004	28.6 ± 9.1	28.8 ± 8.9	0.022
White	32,937 (68.0)	82,351 (71.2)	0.070	32,632 (68.2)	32,476 (67.9)	0.007
Black or African American	4797 (9.9)	7532 (6.5)	0.124	4598 (9.6)	4790 (10.0)	0.013
Unknown race	6152 (12.7)	15,116 (13.1)	0.011	6109 (12.8)	6004 (12.5)	0.007
Factors influencing health status and contact with health services	21,000 (43.3)	44,871 (38.8)	0.093	20,526 (42.9)	20,722 (43.3)	0.008
Comorbidities (ICD-10-CM code)						
Overweight and obesity (E66)	7182 (14.8)	15,151 (13.1)	0.050	7075 (14.8)	6944 (14.5)	0.008
Neoplasms (C00–D49)	6615 (13.7)	13,849 (12.0)	0.050	6410 (13.4)	6381 (13.3)	0.002
Vitamin D deficiency (E55)	6321 (13.0)	14,085 (12.2)	0.026	6200 (13.0)	6151 (12.9)	0.003
Mood disorders (F30–F39)	6297 (13.0)	12,553 (10.9)	0.066	6111 (12.8)	6049 (12.6)	0.004
Sleep disorders (G47)	5167 (10.7)	11,810 (10.2)	0.015	5082 (10.6)	4882 (10.2)	0.014
Dyslipidemia (E78)	3815 (7.9)	11,148 (9.6)	0.062	3783 (7.9)	3660 (7.7)	0.010
Anemias (D64)	3959 (8.2)	5843 (5.1)	0.126	3684 (7.7)	3592 (7.5)	0.007
Diseases of liver (K70–K77)	3199 (6.6)	4214 (3.6)	0.135	2871 (6.0)	2745 (5.7)	0.011
Obstructive sleep apnea (G47.33)	2341 (4.8)	5264 (4.6)	0.013	2312 (4.8)	2189 (4.6)	0.012
Diabetes mellitus (E08–E13)	1956 (4.0)	3878 (3.4)	0.036	1893 (4.0)	1805 (3.8)	0.010
Nicotine dependence (F17)	1847 (3.8)	3100 (2.7)	0.064	1741 (3.6)	1661 (3.5)	0.009
COVID-19 (U07.1)	1530 (3.2)	3520 (3.0)	0.007	1509 (3.2)	1456 (3.0)	0.006
Malnutrition (E40–E46)	1605 (3.3)	1883 (1.6)	0.109	1401 (2.9)	1349 (2.8)	0.007
Ischemic heart diseases (I20–I25)	812 (1.7)	1265 (1.1)	0.050	746 (1.6)	709 (1.5)	0.006
Chronic kidney disease (CKD) (N18)	391 (0.8)	532 (0.5)	0.044	345 (0.7)	336 (0.7)	0.002
Cerebral infarction (I63)	237 (0.5)	440 (0.4)	0.017	227 (0.5)	224 (0.5)	0.001
Hyperparathyroidism and other disorders of parathyroid gland (E21)	192 (0.4)	372 (0.3)	0.012	191 (0.4)	169 (0.4)	0.008
Laboratory data						
Hemoglobin ≥12 g/dL	26,935 (55.6)	66,999 (57.9)	0.047	26,661 (55.7)	26,783 (56.0)	0.005
Hemoglobin A1c ≥7%	1328 (2.7)	2672 (2.3)	0.027	1283 (2.7)	1198 (2.5)	0.011
Albumin g/dL (≥3.5 g/dL)	23,737 (49.0)	54,493 (47.1)	0.038	23,342 (48.8)	23,917 (50.0)	0.024
eGFR >60 mL/min/1.73 m^2^	25,151 (51.9)	56,704 (49.0)	0.058	24,599 (51.4)	24,490 (51.2)	0.005
Vitamin supplementations						
Overall vitamins	11,526 (23.8)	20,664 (17.9)	0.146	11,057 (23.1)	10,910 (22.8)	0.007
Vitamin D	6685 (13.8)	12,696 (11.0)	0.086	6498 (13.6)	6403 (13.4)	0.006
Zinc supplementation	747 (1.5)	1279 (1.1)	0.038	697 (1.5)	688 (1.4)	0.002

BMI = body mass index, eGFR = estimated glomerular filtration rate, ICD-10-CM = International Classification of Diseases, 10th Revision, Clinical Modification, SMD = standardized mean difference, ZD = zinc deficiency.

* SMD values <0.1 indicate adequate balance between groups.

**Figure 1.**
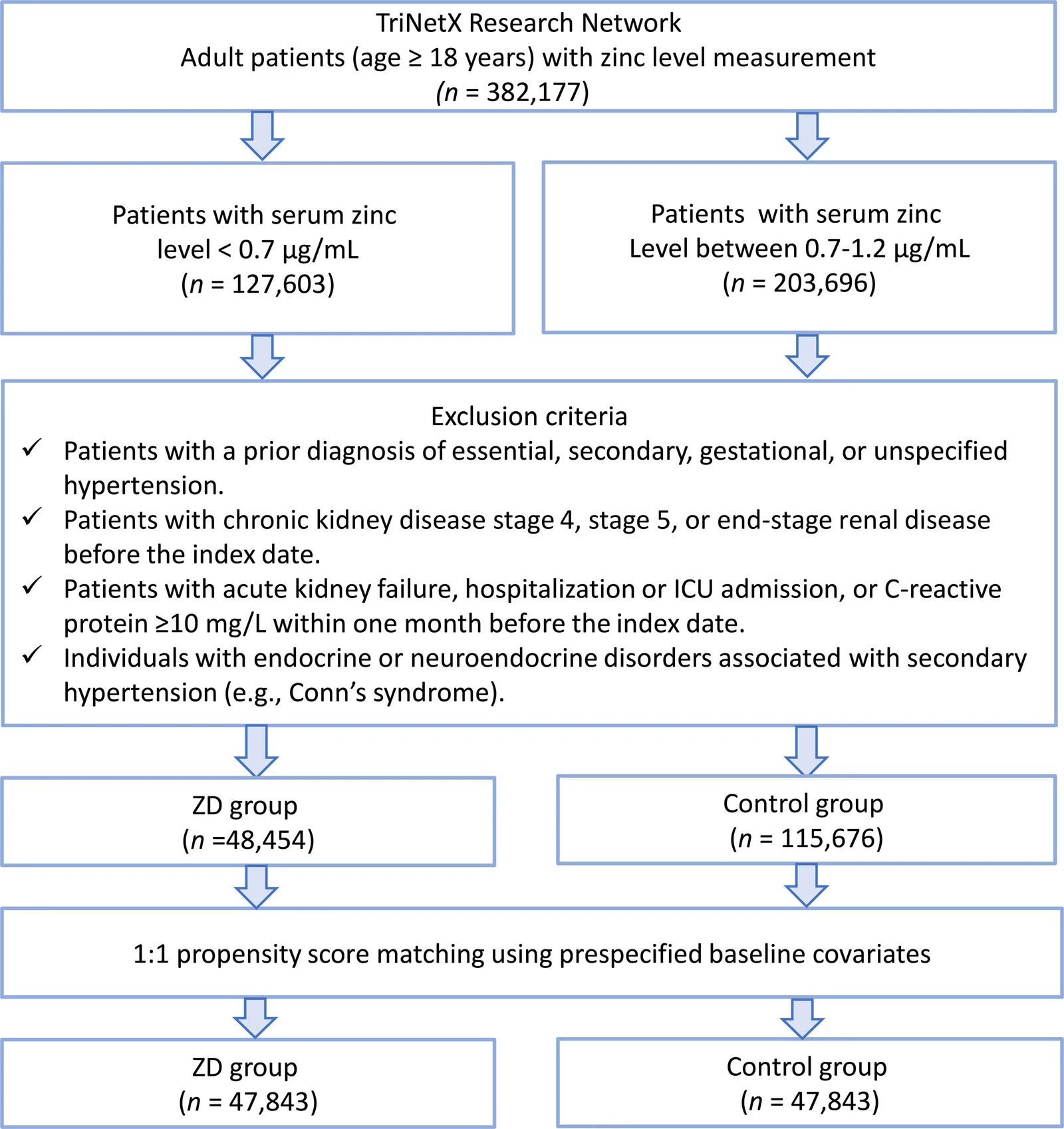
Patient selection and cohort construction. The flowchart illustrates the selection, exclusion, and 1:1 propensity score matching processes used to construct the zinc-deficient and control cohorts from the TriNetX Research Network. ICU = intensive care unit, ZD = zinc deficiency.

**Figure 2.**
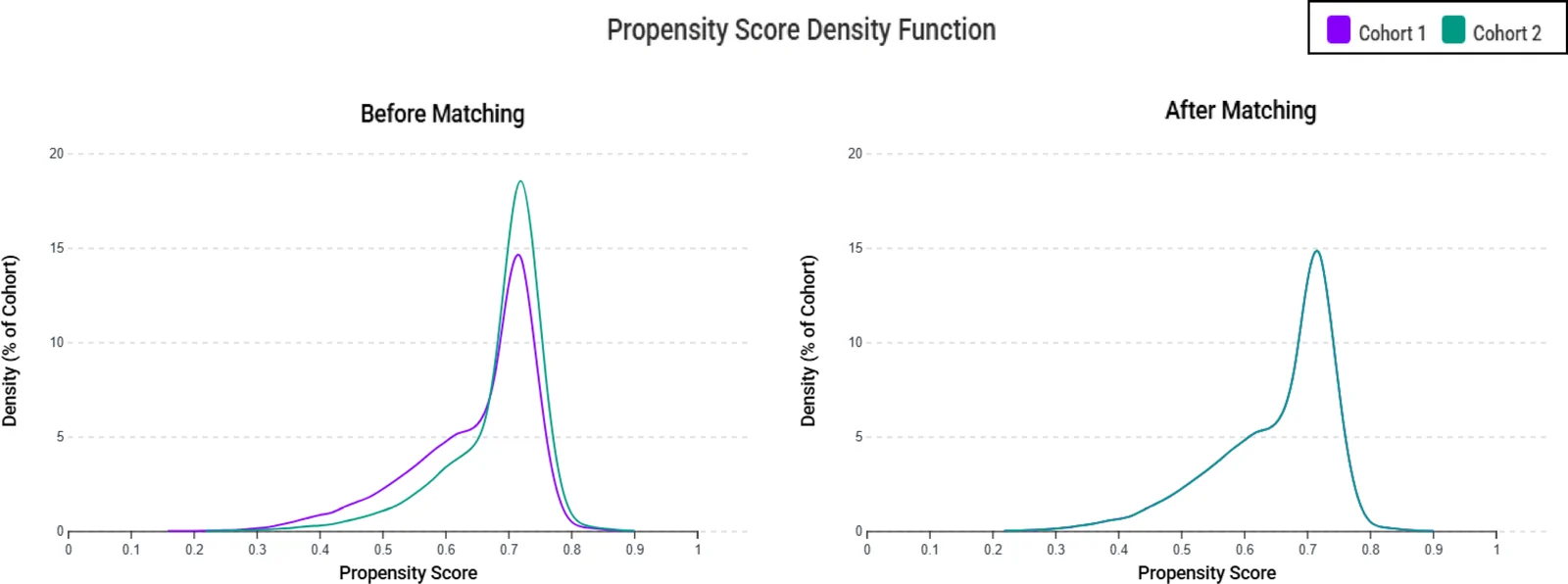
Propensity score distributions before and after matching. Propensity score density distributions are shown for the zinc-deficient cohort (cohort 1) and control cohort (cohort 2). Substantial overlap between the distributions was observed after 1:1 propensity score matching.

### 3.2. Association between zinc deficiency and new-onset hypertension at 5-year follow-up

During 5-year follow-up, zinc deficiency was significantly associated with incident hypertension. Primary hypertension developed in 3614 (7.6%) zinc-deficient patients vs 3218 (6.7%) controls, corresponding to a 20% higher hazard of primary hypertension (HR 1.20; *P* < .001). Zinc deficiency was also associated with a higher hazard of secondary hypertension (HR 1.82; *P* < .001) and hypertension-related cardiac and renal complications (HR 1.69; *P* < .001). In contrast, hypertensive crisis showed a smaller association (HR 1.09; *P* = .028). As a positive control outcome, pneumonia occurred more frequently in the zinc-deficient group (3.6% vs 2.8%; HR 1.36; *P* < .001) (Table 2).

**Table 2:** Association between zinc deficiency and new-onset hypertension at 5-year follow-up.

Outcomes	ZD group (n = 47,843)	Control group (n = 47,843)	HR (95% CI)	*P* value
	events (%)	events (%)		
Primary hypertension	3614 (7.6)	3218 (6.7)	1.20 (1.14–1.25)	<.001
Secondary hypertension	200 (0.42)	116 (0.24)	1.82 (1.45–2.29)	<.001
Cardiac and renal complications*	626 (1.3)	393 (0.82)	1.69 (1.49–1.92)	<.001
Hypertensive crisis	1320 (2.8)	1281 (2.7)	1.09 (1.01–1.18)	.028
Pneumonia (positive control)	1717 (3.6)	1334 (2.8)	1.36 (1.27–1.47)	<.001

CI = confidence interval, HR = hazard ratio, ZD = zinc deficiency.

* Hypertension-related cardiac and renal complications.

### 3.3. Association between zinc deficiency and new-onset hypertension at 3-year and 7-year follow-up

At 3 years, associations were generally similar to or slightly stronger than those at 5 years. The HRs were 1.21 for primary hypertension (*P* < .001) and 2.03 for secondary hypertension (*P* < .001) (Table 3). Cardiac–renal complications (HR 1.66; *P* < .001) and hypertensive crisis (HR 1.13; *P* = .007) also remained significant. At 7 years, most associations were attenuated, with HRs decreasing to 1.17 for primary hypertension and 1.66 for secondary hypertension. Hypertensive crisis was no longer significant (HR 1.05; *P* = .170). Overall, the associations remained directionally consistent across 3-, 5-, and 7-year follow-up, although several estimates weakened with longer follow-up (Table 3).

**Table 3:** Association between zinc deficiency and new-onset hypertension at 3- and 7-year follow-up.

Outcomes	3-yr (n = 47,843 for each group)		7-yr (n = 47,843 for each group)	
	HR (95% CI)	*P* value	HR (95% CI)	*P* value
Primary hypertension	1.21 (1.14–1.27)	<.001	1.17 (1.12–1.22)	<.001
Secondary hypertension	2.03 (1.55–2.66)	<.001	1.66 (1.34–2.04)	<.001
Cardiac and renal complication*	1.66 (1.42–1.94)	<.001	1.56 (1.39–1.74)	<.001
Hypertensive crisis	1.13 (1.03–1.24)	.007	1.05 (0.98–1.13)	.170
Positive control-pneumonia	1.41 (1.29–1.53)	<.001	1.34 (1.25–1.43)	<.001

CI = confidence interval, HR = hazard ratio.

* Hypertension-related cardiac and renal complications.

### 3.4. Dose–response analyses

To assess dose–response associations, 4 zinc concentration categories were compared with the reference range of 0.7 to 1.2 µg/mL (Table 4). Primary hypertension showed a J-shaped pattern: severe zinc deficiency (<0.5 µg/mL) was associated with a 57% higher hazard, moderate deficiency (0.5–0.7 µg/mL) with a 12% higher hazard, and elevated zinc levels (>1.2 µg/mL) with a 12% higher hazard. Pneumonia showed a similar J-shaped pattern.

**Table 4:** Dose–response relationship between serum zinc concentration and hypertension outcomes at 5-year follow-up.

Outcomes	Serum zinc level, HR (95% CI)				Dose–response pattern	J-shaped trend
	<0.5µg/mL (n = 4495)	0.5–0.7 µg/mL (n = 44,542)	0.7–1.2 µg/mL	>1.2 µg/mL (n = 22,776)		
Primary hypertension	1.57 (1.39–1.78)	1.12 (1.06–1.17)	Reference	1.12 (1.04–1.20)	Yes	Yes
Secondary hypertension	5.22 (3.08–8.84)	1.42 (1.10–1.84)	Reference	0.62 (0.40–0.96)	Yes	No
Cardiac and renal complication*	2.89 (2.25–3.71)	1.33 (1.16–1.52)	Reference	0.95 (0.77–1.18)†	Yes	No
Hypertensive crisis	0.93 (0.75–1.14)‡	1.13 (1.04–1.22)	Reference	0.85 (0.76–0.96)	No	No
Positive control-pneumonia	1.90 (1.60–2.26)	1.24 (1.15–1.34)	Reference	1.25 (1.13–1.38)	Yes	Yes

CI = confidence interval, HR = hazard ratio.

* Hypertension-related cardiac and renal complications.

† *P* > .05.

Secondary hypertension showed a different pattern. Severe deficiency was associated with a markedly higher hazard (HR 5.22), while moderate deficiency was associated with a 42% higher hazard. In contrast, elevated zinc levels were associated with a lower observed hazard (HR 0.62), which should be interpreted cautiously given the observational design. For cardiac and renal complications, the hazard increased progressively within the zinc-deficient range, whereas elevated zinc levels were not associated with a higher hazard.

### 3.5. Subgroup analyses by age and sex

Subgroup analyses were conducted by age and sex. Age-stratified analyses (18–50 vs >50 years) showed no significant interactions (Table 5). For primary hypertension, HRs were 1.11 in younger adults (*P* = .024) and 1.15 in older adults (*P* < .001; *P* interaction = 0.517). Secondary hypertension and cardiorenal complications also showed no significant heterogeneity by age. Sex-stratified analyses showed significant effect modification for primary hypertension (Table 6). The association was stronger in men (HR 1.28; *P* < .001) than in women (HR 1.13; *P* < .001; *P* interaction = 0.021). Secondary hypertension showed a similar trend, although the interaction was not significant, while cardiorenal complications were similar between sexes.

**Table 5:** Subgroup analysis of the association between zinc deficiency and new-onset hypertension by age.

Outcomes	18–50 yr (n = 26,459)		>50 yr (n = 21,042)		*P* for interaction
	HR (95% CI)	*P* value	HR (95% CI)	*P* value	
Primary hypertension	1.11 (1.01–1.22)	.024	1.15 (1.09–1.21)	<.001	.517
Secondary hypertension	1.62 (1.06–2.47)	.025	2.10 (1.58–2.79)	<.001	.311
Cardiac and renal complications*	1.98 (1.37–2.85)	<.001	1.53 (1.34–1.75)	<.001	.251
Hypertensive crisis	1.11 (1.01–1.22)	.031	1.05 (0.96–1.15)	.319	.406
Positive control-pneumonia	1.29 (1.16–1.45)	<.001	1.33 (1.21–1.46)	<.001	.682

CI = confidence interval, HR = hazard ratio.

* Hypertension-related cardiac and renal complications.

**Table 6:** Subgroup analysis of the association between zinc deficiency and new-onset hypertension by sex.

Outcomes	Male (n = 11,487)		Female (n = 34,809)		*P* for interaction
	HR (95% CI)	*P* value	HR (95% CI)	*P* value	
Primary hypertension	1.28 (1.17–1.39)	<.001	1.13 (1.07–1.20)	<.001	.021
Secondary hypertension	2.38 (1.60–3.56)	<.001	1.83 (1.35–2.49)	<.001	.342
Cardiac and renal complications*	1.70 (1.39–2.09)	<.001	1.75 (1.47–2.08)	<.001	.833
Hypertensive crisis	1.05 (0.91–1.22)	.486	1.12 (1.02–1.23)	.022	.464
Positive control-pneumonia	1.47 (1.30–1.67)	<.001	1.28 (1.17–1.40)	<.001	.087

CI = confidence interval, HR = hazard ratio.

* Hypertension-related cardiac and renal complications.

## 4. Discussion

In this large-scale, longitudinal cohort study of 95,686 propensity score-matched participants followed for 5 years, zinc deficiency was associated with a 20% higher hazard of new-onset primary hypertension. More pronounced associations were observed for secondary hypertension (HR 1.82) and hypertension-related cardiac and renal complications (HR 1.69). Dose–response analyses revealed a J-shaped relationship, with both severe zinc deficiency (<0.5 µg/mL) and elevated zinc levels (>1.2 µg/mL) associated with increased hypertension risk. The association between zinc deficiency and primary hypertension was significantly stronger in men than in women, while age did not modify these associations. These findings remained consistent across the 3- and 7-year follow-up analyses.

Our finding that zinc deficiency was associated with a 20% higher hazard of incident primary hypertension adds large-scale observational evidence to the literature on micronutrient status and hypertension. This association aligns with mechanistic insights from experimental studies demonstrating that zinc deficiency promotes hypertension through dysregulation of renal sodium handling.^[14,15]^ Our study used standardized outcome definitions and propensity score matching to improve comparability between cohorts. Previous human studies had predominantly cross-sectional or case-control designs with limited sample sizes that could not establish temporal relationships or adequately control for confounders.^[18]^ The observed association is clinically relevant given the global burden of zinc deficiency, which affects over 20% of the population in most low-income and middle-income countries.^[27]^ Whether correction of zinc deficiency through dietary interventions or supplementation can reduce the subsequent risk of hypertension requires evaluation in prospective interventional studies.

The substantially higher hazard of secondary hypertension associated with zinc deficiency (HR 1.82) is a notable finding of our study and has not been previously reported in human cohorts. Secondary hypertension results from identifiable underlying causes, including renal parenchymal disease, endocrine disorders, and renovascular abnormalities.^[28]^ Mechanistically, zinc plays a critical role in multiple systems that are implicated in secondary hypertension. In the kidney, zinc deficiency impairs sodium–chloride cotransporter regulation and epithelial sodium channel function, potentially exacerbating renal parenchymal hypertension through enhanced sodium retention.^[14,15]^ Zinc also modulates the renin–angiotensin–aldosterone system through its effects on angiotensin-converting enzyme activity, which could contribute to both renal and endocrine causes of hypertension.^[29]^ Furthermore, the antioxidant properties of zinc mediated through copper–zinc superoxide dismutase and metallothionein may protect against oxidative stress-mediated vascular dysfunction.^[30,31]^ The stronger association observed for secondary hypertension warrants further investigation into whether zinc status is related to disease-specific pathways underlying secondary hypertension.

Our observation that zinc deficiency was associated with a 69% higher hazard of hypertension-related cardiac and renal complications extends evidence on the cardiovascular and renal correlates of zinc status. No prior cohort study has examined zinc deficiency in relation to specific hypertensive complications. In the cardiovascular system, zinc deficiency promotes oxidative stress, endothelial dysfunction, and pro-inflammatory cytokine activation, all of which contribute to hypertensive heart disease and heart failure development.^[32,33]^ Zinc is essential for maintaining the structural integrity of cardiomyocytes and regulating intracellular calcium homeostasis, with deficiency leading to impaired contractility and increased susceptibility to ischemic injury.^[34]^ In the kidney, chronic zinc deficiency not only perpetuates hypertension through sustained sodium retention but also directly contributes to progressive nephron loss through oxidative damage, tubulointerstitial fibrosis, and impaired repair mechanisms.^[35]^ The observed association between zinc deficiency and hypertension-related cardiac and renal complications may reflect shared vascular, metabolic, or renal pathways; however, the present analysis cannot determine whether these associations are independent of blood pressure elevation.

We observed J-shaped associations between serum zinc concentrations and both primary hypertension and pneumonia. This aligns with a large Chinese cohort demonstrating a similar J-shaped relationship between dietary zinc intake and incident hypertension.^[36]^ More recently, Bahadoran et al reported a nonlinear U-shaped association between serum zinc concentrations and the risk of elevated blood pressure over 9 years, with increased risks observed at both low and high serum zinc concentrations.^[37]^ Our findings extend this evidence using measured serum zinc concentrations in a large multicenter population. At lower zinc concentrations, impaired renal sodium handling, reduced antioxidant capacity, and endothelial dysfunction may contribute to hypertension development,^[14,15,29–31]^ whereas the association at higher concentrations may reflect other mechanisms, supplementation, or underlying clinical factors. Clinically, maintaining zinc status within an adequate physiological range may be preferable to indiscriminate supplementation, although an optimal range cannot be established from observational data. Secondary hypertension exhibited a distinct dose–response pattern, with risk confined primarily to the deficiency range. Notably, elevated zinc concentrations were associated with a lower observed hazard of secondary hypertension (HR 0.62), rather than the J-shaped pattern observed for primary hypertension. This difference may reflect the greater contribution of renal, renovascular, and endocrine pathways, in which zinc deficiency may influence sodium handling, the renin–angiotensin–aldosterone system, and oxidative vascular injury.^[14,15,29–31]^ However, the lower observed risk at higher zinc concentrations should not be interpreted as evidence that supraphysiological zinc is protective, because residual confounding, supplementation patterns, and differences in underlying disease may contribute. Similarly, cardiorenal complications showed a dose-dependent increase limited to the deficiency range, whereas elevated zinc levels were not significantly associated with these complications (HR 0.95).

Our subgroup analyses revealed important effect modifications by sex, but not by age, with implications for targeted prevention strategies. Men with zinc deficiency demonstrated a significantly stronger association with primary hypertension (HR 1.28) than women (HR 1.13) (*P* interaction = 0.021). One possible explanation is the vasculoprotective effect of estrogen, which may enhance endothelial function and favorably influence blood pressure regulation, potentially attenuating the association between zinc deficiency and hypertension in women.^[38]^ However, as this was an observational subgroup analysis, this mechanism remains speculative. Prior studies have also noted sex-specific variations in the metabolic and endocrine effects of zinc.^[39,40]^ These observations collectively support the biological plausibility of sex-based differences in the relationship between zinc status and hypertension risk. We found no statistically significant evidence that the association between zinc deficiency and hypertension differed between the predefined age strata. However, the absence of a statistically significant interaction should not be interpreted as evidence that the magnitude of the association is identical across all age groups.

This study has several limitations. First, despite propensity score matching, residual and unmeasured confounding remains possible because dietary zinc intake, dietary patterns, physical activity, socioeconomic factors, and other lifestyle characteristics are not consistently captured in electronic health records. Although we matched nutritional and metabolic indicators, including body mass index, malnutrition, serum albumin, major comorbidities, and vitamin supplementation, these variables cannot fully substitute for direct assessment of diet and lifestyle. Second, zinc levels were assessed at a single time point, which does not account for longitudinal fluctuations, duration of deficiency before measurement, or temporal trends in zinc status. In addition, fasting status and the exact timing of blood collection were not consistently available, although serum zinc concentrations may vary according to recent food intake and sampling time. This may have resulted in some exposure misclassification. Third, TriNetX relies on routinely collected clinical data from multiple healthcare organizations. Although participating organizations map their data to standardized terminologies and common data models, differences in laboratory procedures, diagnostic coding, documentation practices, healthcare utilization, patient populations, and completeness of clinical information may introduce inter-institutional heterogeneity and potential exposure or outcome misclassification. Because our analysis used aggregated network-level data, we could not quantify between-center heterogeneity. Fourth, we could not assess dietary zinc intake or bioavailability, or differentiate primary nutritional deficiency from deficiency secondary to malabsorption or pathological losses. Fifth, information on zinc supplementation may be incomplete in electronic health records, and we could not account for over-the-counter use. Finally, although we excluded participants with acute illness and matched on multiple comorbidities, we cannot entirely exclude the possibility that low serum zinc serves partly as a biomarker of poor nutritional status, chronic disease burden, or other underlying health conditions rather than representing a direct causal factor for hypertension.

## 5. Conclusion

This large-scale retrospective cohort study found that zinc deficiency was associated with a higher hazard of incident hypertension after propensity score matching, with particularly strong associations observed for secondary hypertension and hypertension-related cardiac and renal complications. The nonlinear dose–response pattern suggests that both low and elevated serum zinc concentrations may be associated with primary hypertension; however, an optimal therapeutic zinc range cannot be established from these observational data. These findings support further evaluation of serum zinc status as a potential risk marker and provide a rationale for prospective studies, including randomized controlled trials, to determine whether correction of zinc deficiency can modify hypertension risk and related cardiovascular outcomes.

## Author contributions

**Conceptualization:** Kuo-Chuan Hung, Shu-Wei Liao, Yi-Chen Lai, I-Wen Chen.

**Data curation:** Kuo-Chuan Hung, Shu-Wei Liao, Yi-Chen Lai, I-Wen Chen.

**Formal analysis:** Kuo-Chuan Hung, Shu-Wei Liao, Yi-Chen Lai, I-Wen Chen.

**Investigation:** Hsiu-Lan Weng, Ming-Chung Lin.

**Methodology:** Hsiu-Lan Weng.

**Software:** Kuo-Chuan Hung, Hsiu-Lan Weng.

**Supervision:** Kuo-Chuan Hung.

**Validation:** Kuo-Chuan Hung, Shu-Wei Liao, Yi-Chen Lai, Ming-Chung Lin, I-Wen Chen.

**Visualization:** Kuo-Chuan Hung, Yi-Chen Lai.

**Writing – original draft:** Kuo-Chuan Hung, I-Wen Chen.

**Writing – review & editing:** Kuo-Chuan Hung, I-Wen Chen.
